# Technique for insertion of a Scheker prosthesis for failed Sauve-Kapandji with a well fixed ulnar stem: A case report^☆^

**DOI:** 10.1016/j.ijscr.2023.108913

**Published:** 2023-10-05

**Authors:** Adam Haydel, Nathan Redlich, Jared Smith, Rasheed Ahmad

**Affiliations:** a LSUHSC New Orleans, Department of Orthopaedic Surgery, 2021 Perdido Street, 7th floor, New Orleans, LA 70112, USA; b LSUHSC Shreveport, 1501 Kings Highway, Shreveport, LA 71103, USA; c Baton Rouge, Orthopaedic Clinic, 8080 Bluebonnet Blvd, Ste 1000, Baton Rouge, LA, 70810, USA

**Keywords:** DRUJ, Osteotomy, Sauve-Kapandji, Scheker prosthesis

## Abstract

**Introduction:**

The Scheker prosthesis is a distal radioulnar joint (DRUJ) arthroplasty used as a salvage option for many DRUJ pathologies.

**Presentation of case:**

We report the case of a patient who underwent insertion of a Scheker prosthesis for continued pain and limited motion at the wrist in the setting of a failed Sauve-Kapandji with a well fixed ulnar stem and DRUJ pseudo-arthrosis.

**Discussion:**

This report aims to provide a technique for ulnar stem removal without compromising the bone needed for the Scheker prosthesis and for describing the location of a DRUJ osteotomy without compromising radio-lunate stability.

**Conclusion:**

The Scheker prosthesis is able to be safely inserted for DRUJ salvage after removal of a well fixed ulnar stem if careful removal prevents destruction of the ulna, as described here.

## Highlights

• Scheker prosthesis can be used as a salvage option for many DRUJ pathologies.
• Removal of a well fixed ulnar stem may require osteotomy and use of Kirchner wires.
• The ulnar osteotomy should not take more bone than can be replaced with prosthesis.
• Use fluoroscopy to plan DRUJ osteotomy for a pseudoarthrosis

## 1 Introduction

The distal radioulnar joint (DRUJ), which consists of the ulnar seat articulating with the sigmoid notch of the radius, not only functions in forearm pronation and supination, but also sustains transverse stress with lifting and axial stress with gripping [1]. The ulna acts as a fixed post around which the radius rotates. Removal of the distal ulna can result in instability and pain from the ulnar stump impinging on the radius. Many pathologies of the DRUJ have been described, including arthritis (posttraumatic, degenerative, rheumatoid, and inflammatory), tumors, congenital anomalies, and instability. Previous treatment options have mostly consisted of fusion or excision. Multiple procedures have been described for chronic DRUJ instability or arthritis, including total ulnar head excision (Darrach procedure), DRUJ arthrodesis with ulnar shaft resection and creation of a distal ulnar pseudo-arthrosis (Suave-Kapandji), hemiresection interposition, and matched ulnar resection. [[2], [3], [4]]

The Scheker prosthesis (Aptis Medical, Louisville, KY) was developed to better address symptomatic post-traumatic, inflammatory, and degenerative DRUJ arthritis and instability, as well as failed Darrach, Sauve-Kapandji, or hemiresection interposition arthroplasties [5,6]. Cleared by the Federal Drug Administration (FDA) in 2005, it replaces the ulnar head and sigmoid notch with a semiconstrained modular implant using an intramedullary ulnar stem, a radial sided plate, and an ultra-high molecular weight polyethylene (UHMWPE) ball [5,6].

We describe a technique for implanting a Scheker prosthesis in a patient with pseudo-arthrosis of the head of the ulna to the radius and a well fixed ulnar stem after a failed Sauve-Kapadji procedure. This case is of note because it describes our technique for safe explantation of the well fixed ulnar prosthesis without ruining the ulna as well as the location of the DRUJ osteotomy, while avoiding radio-lunate instability. This case report was written in line with the Surgical Case Report (SCARE) criteria [7].

## 2 Presentation of case

A 44-year-old right hand dominant male welder presented to the senior author with a primary complaint of worsening sharp pain in the right forearm with activities and pain in the elbow with pronation and supination. He reported a work injury to the right arm in 2006 and subsequently underwent multiple surgeries. In addition, he reported right long finger numbness at night and small finger numbness during the day. He had a well healed scar on the ulnar side of the wrist and a normal motor and sensory exam, with 2-point discrimination 5 mm in all digits. He was point tender about 2 cm (cm) proximal to the ulnar head, along the ulnar side of the wrist. Range of motion (ROM) at the right elbow was +5–140°, and range of motion at the wrist was 30° extension, 60° flexion, 30° of pronation, and 20° of supination.

**Fig. 1:**
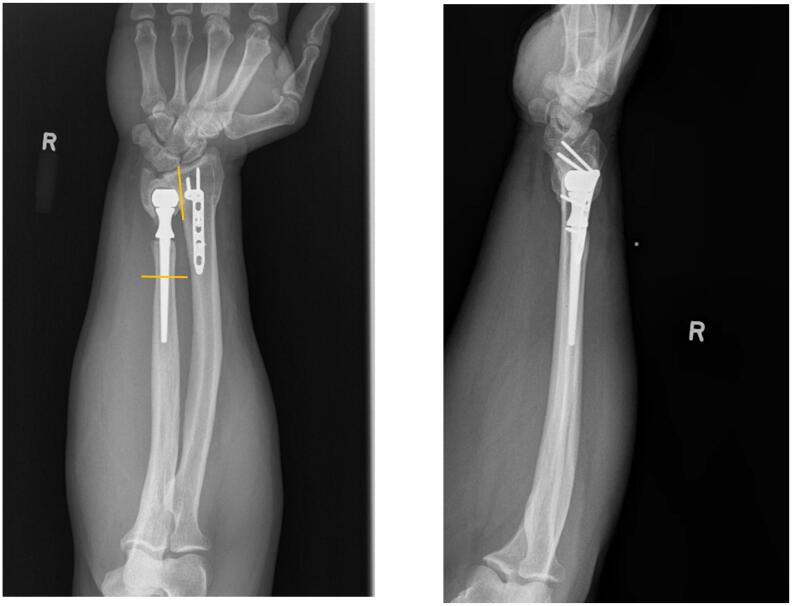
Anteroposterior (AP) and lateral radiographs of the patient's right forearm at presentation, demonstrating the pesudo-arthrosis of the distal ulna to the radius and ulnar stem within the head and diaphysis of the ulna. Some lucency can be seen around the ulnar head but the stem appears well fixed. Lines demonstrating approxiate location of osteotomies can be seen on AP radiograph.

**Fig. 2:**
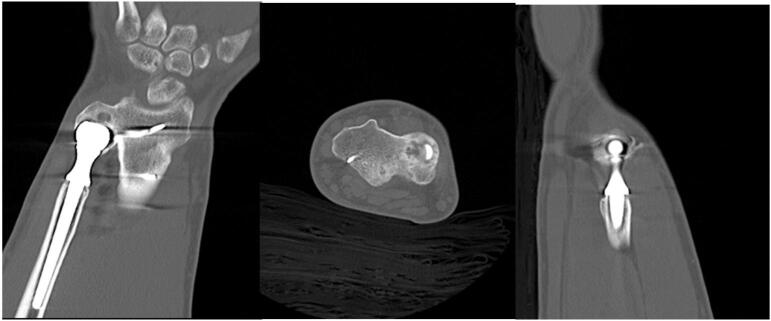
Coronal, axial, and sagittal cuts from the wrist computed tomography (CT) scan showing the pseudo-arthrosis of the distal ulna with the radius and the well fixed ulnar stem.

Anteroposterior (AP), lateral, and oblique radiographs were obtained in clinic that demonstrated a plate along the dorsal midline radius, an ulnar prosthesis inside the head of the ulna with a stem in the ulnar shaft, and lucency around the distal prosthesis inside the ulnar head (Fig. 1). His erythrocyte sedimentation rate (ESR) was 2, C-reactive protein (CRP) was 4.3, and white blood cell (WBC) count was 10.3. A computed tomography (CT) scan was obtained and showed bony ingrowth into the stem of the ulnar prosthesis (Fig. 2). Discussion was had with the patient and it was determined that the best salvage option would be insertion of a Scheker prosthesis.

The dorsal ulnar scar was excised and the dorsal ulnar sensory nerve was protected. The extensor tendons were exposed and a radially based flap of extensor retinaculum was made off the 5th and 6th extensor compartments. The head of the ulna and pseudoarthrosis was exposed. The dorsal radial plate was removed. Under fluoroscopy, a saw was used at the ulna-radius junction to create an osteotomy in the pseudo-arthrosis while maintain radio-lunate stability. The ulnar head was removed from around the prosthesis using a rongeur and osteotomes. In order to remove the well fixed ulnar stem, an osteotomy was made with a saw and osteotomes, creating approximately a 4 centimeter gap. The osteotomy was made to remove as much of the bony ingrown ulna as could be replaced by the Scheker prosthesis. Kirchner wires (K wires) were driven along the junction of the ulnar stem and bone until the prosthesis was loose in order to preserve as much ulna as possible.

The radial plate trial was placed in appropriate position and the pegs were drilled. The final radial plate was placed with the compression screw and locking screws under fluoroscopy. The ulna was reamed to appropriate size and the implant was placed. The extensor flap was brought under the extensor digiti minimi (EDM) and extensor carpi ulnaris (ECU) tendons to prevent direct contact with the plate. A drain was placed and the wound was closed in standard fashion after copious irrigation. The operative extremity was placed in a long arm splint.

**Fig. 3:**
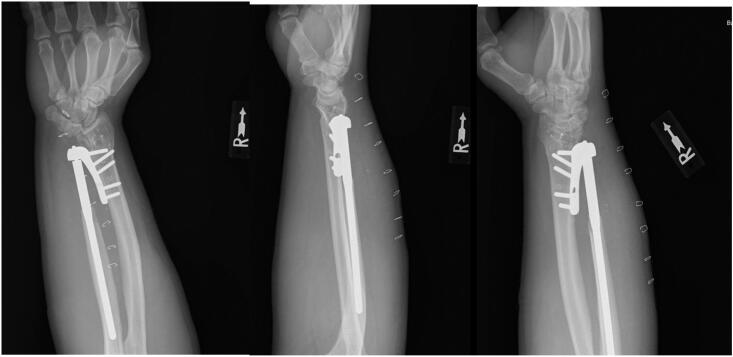
Anteroposterior (AP), lateral, and oblique 2 week post-operative radiographs showing the Scheker prosthesis. The site of the DRUJ osteotomy is seen.

**Fig. 4:**
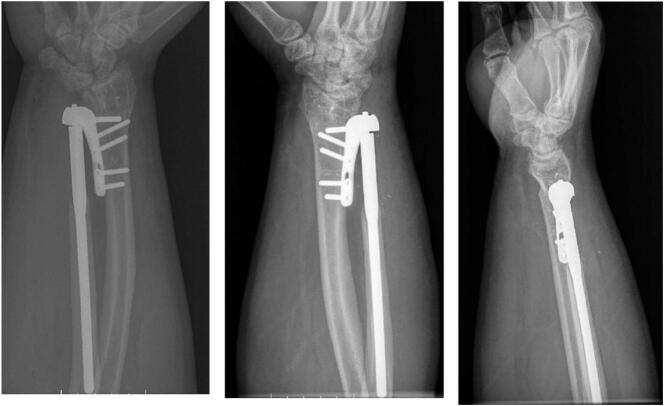
Anteroposterior (AP), lateral, and oblique 6 month post-operative radiographs showing the Scheker prosthesis in place without evidence of loosening or hardware complication. The site of the DRUJ osteotomy is seen without evidence of radio-lunate instability.

At 2 weeks post-op, the patient had mild pain over the dorsal ulnar hand. His wound was well healed and he demonstrated smooth pronation, supination, flexion and extension of the wrist. He had no complaints in the radio-lunate articulation or radial instability of the lunate on the radius (Fig. 3). The staples were removed and he was continued in the long arm splint but allowed to work on wrist flexion and extension. He had the splint exchanged for a brace around 3 weeks postop. At 6 weeks postop he had no pain, 50° of wrist extension, 40° of wrist flexion, 45° of pronation, and 80° of supination. AP, lateral, and oblique radiographs showed appropriate position of the implant without any complications. His brace was removed and a 20-pound lifting restriction was given to the patient. At 6 months he noted some mild pain at night and his physical exam remained unchanged from his previous visit (Fig. 4). Overall, he expressed satisfaction with his outcome and functionality with the arm. Written informed consent was obtained from the patient for publication of this case report and accompanying images. The senior author is a fellowship-trained hand surgeon with 20 years of private practice experience. The surgical technique for the Shecker prosthesis is well-written and accurately details the surgery and perils.

## 3 Discussion

In this article, we present a technique for performing a DRUJ arthroplasty with a Scheker prosthesis in the setting of a failed Sauve-Kapandji with a DRUJ pseudo-arthrosis and a well fixed ulnar stem. The implants that the patient presented with have been described in the European literature [8]. One of the technical challenges in this case is deciding where to make the DRUJ osteotomy. As can be seen in Fig. 1, Fig. 2, the remaining head of the ulna was pseudo-arthrosed to the radius. In this case, fluoroscopy was used to assist in finding the DRUJ to create an osteotomy at the sigmoid notch. The most difficult part was finding the site for the osteotomy through the head of the ulna. The decortication of the sigmoid notch of the radius led to falsely following the contour of the radius and ulna in making the osteotomy. That is what put the osteotomy approximately 2 mm into the lunate fossa. This is a critical step due to the risk of radio-lunate instability if too much radius is resected.

The other technical challenge we describe is removing the well fixed ulnar stem without damaging the ulna. Approximately 2 cm of the distal ulna was resected with a saw and osteotomes prior to trying to remove the well-fixed ulnar stem for two reasons. It gave us less bone from which we had to dislodge the stem. The resection also resulted in a shorter distance along that stem requiring K-wire penetration through the healing bone. 2 cm was chosen as the length of ulna to remove because the Shecker prosthesis would still be able to reconstruct that deformity. This ended up creating approximately a 4 cm gap, and to accommodate this gap the radial plate was placed slightly more proximal.

Previous case series have described results of the Scheker prosthesis, and some of the studies report failed Sauve-Kapandji as an indication in their cohort [5,9,10]. The literature supports use of this implant as a salvage procedure with overall good results. Axelsson et al. reported significant improvements in pain and Disabilities of the Arm, Shoulder, and Hand questionnaire (DASH) scores and non-significant improvement in grip strength in 9 patients, 2 for failed Sauve-Kapandji, with minimum 2 year follow up [9]. They also reported a median improvement of 10° in forearm rotation. Our patient obtained a 15° improvement in pronation and 60° improvement in supination. One of the larger case series reported included 35 patients. Patients had undergone a total of 56 prior surgical procedures, with 2 being a failed Sauve-Kapandji. They found no significant difference in lifting capacity between the surgical arm and contralateral arm in neutral, supination, or pronation. Patients were satisfied, had improved pain, and improved activities of daily living [5].

Other case series have shown nonsignificant improvements in wrist ROM, decreased pain, and similar grip strength [10,11], improved pain scores and supination strength [1]; in addition to improvements in activities of daily living, strength, and lifting capacity [12].

Reported complication rates after Scheker prosthesis insertion range from 13 to 44 % [1,5,9,10,12]. Some reported complications include transient carpal tunnel syndrome, De Quervain tenosynovitis, lateral elbow pain, bone resorption around radial component screw tip, implant loosening or malposition, fracture, fifth and sixth dorsal compartment tendonitis, heterotopic bone formation, wound complications, infection, and tendon rupture [1,9,10,12].

## 4 Conclusion

This article describes the presentation of a failed Sauve-Kapandji with a well fixed ulnar stem and surgical technique for hardware removal and insertion of a Scheker prosthesis. In this case, a DRUJ osteotomy had to be performed in order to remove the remaining ulnar head which had formed a pseudo-arthrosis with the radius and we describe a technique for finding the best location for the osteotomy while maintaining radio-lunate stability. We also describe our method of removing the well fixed stem without damaging the ulna beyond salvage.

## CRediT authorship contribution statement

Adam Haydel – study concept, data collection, writing, editing

Nathan Redlich – writing, editing

Jared Smith – writing, editing

Rasheed Ahmad – study concept, data collection, writing, editing

## Consent

Written informed consent was obtained from the patient for publication of this case report and accompanying images.

## Registration of research studies

1. Name of the registry: N/A.
2. Unique identifying number or registration ID: N/A.
3. Hyperlink to your specific registration (must be publicly accessible and will be checked): N/A.

## Ethical approval

Case reports do not require IRB ethics approval at our institution. They do not get submitted

to the committee and are exempt.

## Funding research

There is no funding.

## Guarantor

Rasheed Ahmad

## Declaration of competing interest

No benefits in any form have been received or will be received related directly or indirectly to the subject of this article.
